# Diagnostic Performance and Discordance of Kato–Katz Method, POC-CCA Test, and PCR in Detecting *Schistosoma mansoni* in a Low-Prevalence South African Setting

**DOI:** 10.3390/diseases14050164

**Published:** 2026-05-08

**Authors:** Maryline Vere, Wilma ten Ham-Baloyi, Lucy Ochola, Opeoluwa Oyedele, Lindsey Beyleveld, Siphokazi Tili, Takafira Mduluza, Paula Melariri

**Affiliations:** 1 Department of Environmental Health, Faculty of Health Sciences, Nelson Mandela University, Summerstrand, Gqeberha 6001, South Africa; 2 Department of Nursing Science, Faculty of Health Sciences, Nelson Mandela University, Summerstrand, Gqeberha 6001, South Africa; 3 Department of Tropical and Infectious Diseases, Kenya Institute of Primate Research, Nairobi 00502, Kenya; 4 Department of Computing, Mathematical and Statistical Sciences, School of Science, University of Namibia, Windhoek 9000, Namibia; 5 Department of Medical Laboratory Science, Faculty of Health Sciences, Nelson Mandela University, Summerstrand, Gqeberha 6001, South Africa; 6 Department of Biochemistry and Biotechnology, University of Zimbabwe, Harare P.O. Box MP 167, Zimbabwe

**Keywords:** PCR, Kato–Katz technique, prevalence, school-going children, NMB, schistosomiasis, CCA test

## Abstract

Background/Objectives: Intestinal schistosomiasis caused by *Schistosoma mansoni* is often underestimated in low-transmission settings due to the limited sensitivity of traditional stool microscopy. More sensitive approaches, including antigen detection and molecular diagnostics, are required to detect infections where egg excretion is low or intermittent. This study aimed to determine the prevalence of *S. mansoni* infection among school-going children in Nelson Mandela Bay (NMB) using a multi-modal diagnostic approach. Methods: This cross-sectional study included 759 schoolchildren aged 5–14 years from 15 primary schools in NMB. Stool samples were analyzed using the Kato–Katz technique to detect *S. mansoni* eggs, while urine samples were tested using the point-of-care circulating cathodic antigen (POC-CCA) assay for antigen detection. A subset of stool samples from POC-CCA-positive participants (*n* = 28) was further analyzed using conventional PCR (cPCR), targeting the *S. mansoni* cox1 gene, for molecular confirmation. Only a single stool specimen was collected per participant. Results: Among the 759 participants (58% male, 42% female), no egg-positive cases were detected. However, POC-CCA testing identified *S. mansoni* antigen in 3.2% of participants. Of the 28 POC-CCA-positive samples analyzed by cPCR, 9 (32.1%) were PCR-positive, representing molecular confirmation within the antigen-positive subset rather than overall prevalence. Conclusions: Traditional microscopy underestimated *S. mansoni* prevalence in this low-prevalence setting. Antigen detection combined with molecular diagnostics improved case identification and highlighted ongoing transmission. These findings support the integration of sensitive diagnostic tools into schistosomiasis surveillance and control strategies in South Africa.

## 1. Introduction

Schistosomiasis, or bilharzia, is a parasitic disease affecting approximately 230 million people worldwide. In sub-Saharan Africa, *Schistosoma* (*S.*) *mansoni* is a major cause of intestinal schistosomiasis [1], and in South Africa, it significantly contributes to the disease burden in endemic regions [2,3,4]. Clinical manifestations include hepatosplenomegaly, pyrexia, abdominal pain, and haematochezia [5].

The life cycle of *Schistosoma* involves freshwater snails as intermediate hosts, which release cercariae into the water. Human infection occurs when cercariae penetrate the skin during water contact [5]. The parasites migrate through the vasculature, mature into adult worms, and reside in the mesenteric veins, where females produce eggs excreted in feces. In chronic infection, retained eggs trigger granulomatous inflammation and fibrosis, leading to morbidity [6]. Disease pathogenesis is driven by the host immune response to trapped eggs, which also results in intermittent and low egg excretion, complicating diagnosis in low-intensity infections. Sensitive diagnostic tools are therefore essential for early detection, particularly among school-aged children who are most at risk.

Accurate estimation of *Schistosoma mansoni* prevalence in low-transmission settings remains a diagnostic challenge [7]. Traditional stool microscopy (Kato–Katz) has high specificity but limited sensitivity in low-intensity infections due to intermittent and low egg excretion [8]. More sensitive tools, including the point-of-care circulating cathodic antigen (POC-CCA) test and molecular methods such as conventional PCR (cPCR), can detect infections missed by microscopy [9]. However, the POC-CCA test raises specificity concerns in low-prevalence areas, particularly with ‘trace’ results, while cPCR is resource-intensive. The optimal diagnostic strategy for surveillance in very low-endemic settings remains unclear [10,11,12,13].

In South Africa, the Nelson Mandela Bay (NMB) metropolitan area has been considered low-transmission for *S. mansoni* based on a single prior mapping study that reported 0.9% prevalence using Kato–Katz [14]. Critically, that study was conducted in only one town within NMB and did not survey the entire metropolitan area [14]. To date, no study has ever determined the prevalence of *S. mansoni* across the whole of NMB, an area comprising five towns (KwaNobuhle, Motherwell, Ibhayi, Kariega, and Despatch), each with different water bodies, snail habitats, and potential transmission risks.

This study is therefore the first to determine *S. mansoni* prevalence across the entire NMB metropolitan area. It had two aims: (1) to conduct the first area-wide survey covering all five towns; and (2) to establish true prevalence using a sequential diagnostic approach (Kato–Katz → POC-CCA → cPCR). This design allowed us to: (a) assess whether the ‘low-prevalence’ designation holds when sensitive methods are applied area-wide; (b) evaluate the utility of POC-CCA in this setting; and (c) confirm positive antigen results with molecular testing. We hypothesized that sensitive diagnostics would reveal ongoing transmission not detectable by microscopy alone, and that prevalence would vary across towns based on local exposure risks.

## 2. Materials and Methods

### 2.1. Study Area

**Figure 1:**
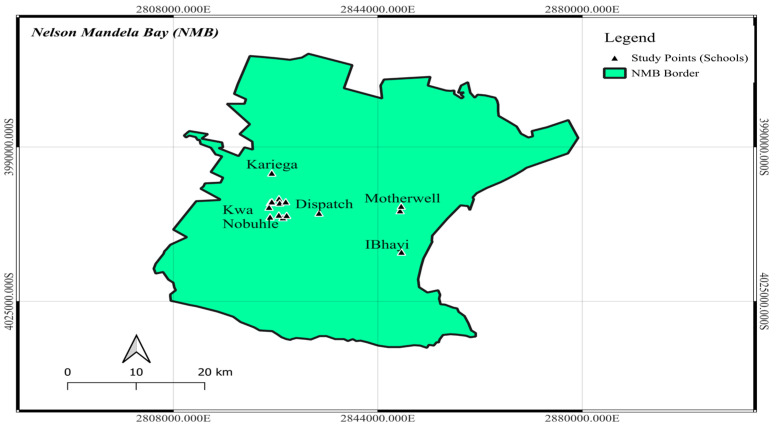
Map showing study area and data collection points (Source: map was generated by researcher using QGIS 3.34 Prizren).

The study was conducted in NMB, which is situated in South Africa’s Eastern Cape province. The study was carried out in five towns within NMB: Motherwell, Despatch, KwaNobuhle, Kariega, and Ibhayi (see Figure 1). The study sites were chosen because they are near dams, rivers, marshes, and streams which are schistosomiasis exposure sites [15]. NMB covers an area of approximately 1959 km^2^, located at the coordinates 33°57′ S, 25°36′ E. As reported by [16], the total population of NMB for that year was 1.33 million individuals. The age group 0–9 constitutes 20% of the overall population, while the following age group closely trails, with a representation of 18% [16]. NMB undergoes prolonged, cool winters from May to September; short, warm summers from December to March; and dry, clear, and windy weather conditions [17]. The average temperature recorded in 2023 was 22.7 °C. In 2023, the highest temperature observed was 35.5 °C in April, whereas the lowest temperature noted was 12 °C in July. NMB receives an average annual precipitation of 331 mm, distributed evenly throughout the year. The range of precipitation varies, with a low of 3.4 mm recorded in August and a peak of 50 mm observed in April.

### 2.2. Study Design

This study employed a quantitative descriptive cross-sectional design to assess the prevalence of *S. mansoni* among schoolchildren in grades 0–7, aged 5 to 14 years, at selected primary schools in Kariega, KwaNobuhle, Motherwell, Ibhayi, and Despatch within the NMB region, and was conducted from August 2023 to February 2024. The study sites were purposefully selected due to the existence of water bodies in residential areas that may act as potential exposure sites for schistosomiasis [18]. Quantitative data was collected to evaluate the prevalence of intestinal schistosomiasis and the diagnostic efficacy of the Kato–Katz technique, the POC-CCA test and cPCR in the low-prevalence area of NMB.

### 2.3. Recruitment Criteria

After obtaining ethics approval and authorization from the Eastern Cape Department of Education (ECDOE), permission was requested from 21 schools to participate in the study. Seventeen schools agreed, while four declined participation through their governing boards. Meetings were held with parents at these schools to explain the study. However, parental consent could not be obtained from two schools, leaving 15 schools eligible. A total of 1500 consent forms were distributed, and 823 signed forms were returned, meeting the criteria for participation.

Participants were selected using a stratified cluster sampling method across 15 primary schools in five towns within NMB. During school visits, the study’s objectives and procedures were clearly explained to principals, teachers, and students. Class rosters of children aged 5–14 years in grades 0–7 were compiled, including names, gender, and age, focusing only on those with parental consent and who provided assent.

The study was communicated in simple language to ensure participant understanding. Participation was voluntary, and students could withdraw at any time. Assent was obtained both verbally and in writing, with parental or guardian consent required. Eligibility criteria included enrolment in one of the selected schools, age between 5 and 14 years, written parental consent, verbal and written assent, fluency in IsiXhosa or Afrikaans, and presence on the sample collection day.

### 2.4. Population and Sampling

In South Africa, the public primary education system is divided into two main stages: the foundation phase, which includes grades R (0) to 3, and the intermediate phase, covering grades 4 to 7. All primary schools within the designated study area that included these grade levels were eligible for participation. A stratified cluster-sampling approach was employed to select study participants. Students were categorized into two groups based on their grade levels (0–3 and 4–7), and a random selection of classes from each grade across all schools was conducted to ensure a representative sample.

**Figure 2:**
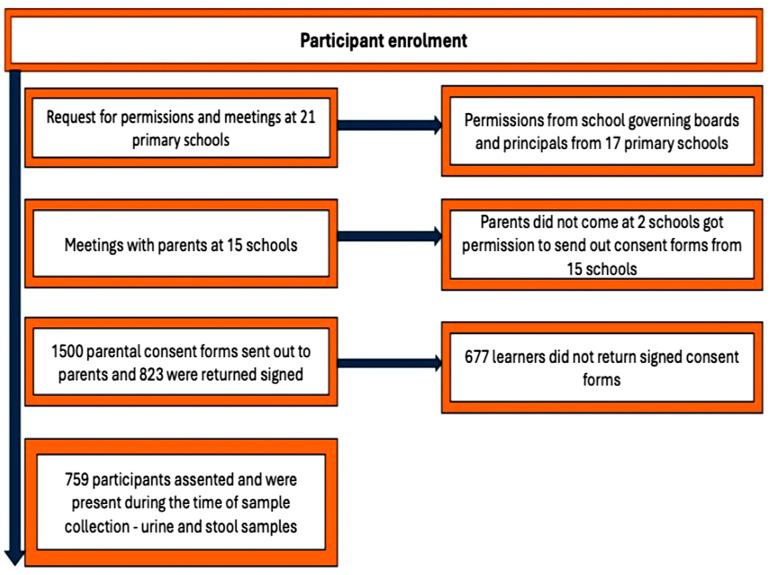
Schematic diagram for study participants’ recruitment, enrolment and participation.

This sampling strategy ensured that every child had an equal probability of selection while maintaining proportional representation of gender and grade distribution in alignment with the broader population. Participation was restricted to students with parental consent. During data collection, 64 potential participants were absent and were therefore excluded. The final study sample consisted of 759 participants, as illustrated in Figure 2.

### 2.5. Sample Size Calculation

This survey was part of a larger study aimed at determining the prevalence of schistosomiasis in Nelson Mandela Bay. The sample size was determined based on the reported 2% prevalence of *S. haematobium* from a previous study in the area [14]. A total of 1972 primary school children participated, with 48% male and 52% female [14]. These prevalence figures formed the basis for calculating the sample size used in this current study, ensuring that the study design was appropriately powered to detect schistosomiasis infections in the target population. Using this prevalence, the formula from [19] was used to calculate the appropriate sample size in the prevalence study as follows:

$$n=\frac{Z^{2}\times p\times (1-p)}{d^{2}}$$

where n= sample size, Z=1.96 representing a confidence level of 95% p is the prevalence of schistosomiasis (using 2% prevalence for schistosomiasis in Nelson Mandela Bay [14]), and d=1% is the precision error.

That is,

$$n=\frac{1.96^{2}\times 0.02\times (1-0.02)}{0.01^{2}}≅753.$$

Adjusting for a 10% nonresponse rate (due to absenteeism or refusal), 753 × 0.1 = 75 additional respondents were required. This yields a minimum sample size of 753, and a maximum of 828 respondents to be considered for this study.

### 2.6. Urine and Stool Sample Collection

A total of 759 urine and stool samples were systematically collected from schoolchildren aged 5 to 14 years across the participating schools. Two 50 millilitre (ml) plastic bottles, characterized by a wide mouth and a screw cap, were supplied to the selected participants. Each bottle was labelled and featured a distinct unique identifier (ID) [20]. Midstream urine samples were collected from 10:00 to 14:00, aligning with the peak period of egg excretion in individuals infected with *Schistosoma* [21]. The timing corresponds with the circadian rhythms of the parasites, thereby facilitating optimal conditions for both egg release and excretion [22]. The researcher performed a visual demonstration to illustrate the correct method for collecting samples and transferring them into specific individual bottles. Participants were instructed to return with containers that held more than half of a urine sample. Samples were gathered from staff bathrooms at the schools and then transported to a private room to maintain participant confidentiality. Participants received soap to facilitate comprehensive handwashing following the return of the sample containers. The plastic bottles containing stool and urine samples were securely sealed and placed in a cooler box before being transported to the laboratory located at Nelson Mandela University for analysis [18]. The average duration for transport from the sample collection site to the laboratory was one hour. In the laboratory, parasitological analysis and the POC-CCA test were conducted on the same day, by the researcher alongside a laboratory technician. Urine samples were collected for POC-CCA testing to detect the *S. mansoni* circulating antigen. Urine sedimentation for *S. haematobium* egg detection was not performed, as the primary objective of this study was to assess *S. mansoni* prevalence.

### 2.7. Kato–Katz Technique

Duplicate Kato–Katz thick smears were prepared from each stool sample using the Helmintex^®^ Kato–Katz Kit (Bio-Manguinhos, Oswaldo Cruz Foundation, Rio de Janeiro, Brazil) according to the manufacturer’s instructions. As only a single stool sample was collected, inter-day variation in egg excretion may have reduced sensitivity for detecting low-intensity infections. Samples were sieved, placed on slides using a template, covered with glycerol–malachite green-soaked cellophane, and compressed for even distribution.

Slides were examined under a Motic B1 Series light microscope at ×10 and ×40 magnifications by two experienced technicians. For quality control, 10% of slides were re-examined by a lead technician. Egg counts were multiplied by 24 to estimate eggs per gram (EPG). Discrepant results or counts differing by more than 20% were re-evaluated to ensure accuracy.

### 2.8. POC-CCA Test

Urine samples were analyzed for *S. mansoni* circulating cathodic antigen using POC-CCA test kits according to the manufacturer’s guidelines (Rapid Medical Diagnostics, Pretoria, South Africa). After equilibrating reagents to room temperature, two drops of urine (≈40 μL each) were added to the cassette well and incubated for 20 min. Results were independently read by two trained technicians and classified as negative, trace, 1+, 2+, or invalid (no control line). Trace results were considered positive, and readings after 25 min were deemed invalid and repeated.

### 2.9. Molecular Examination for Schistosome Infection

cfDNA analysis was performed as an alternative molecular approach for detecting *Schistosoma* infection in samples with low or absent egg excretion but that tested positive for *Schistosoma* antigen via POC-CCA test. cfDNA originates from apoptotic and necrotic parasite cells circulating in human biological fluids, including plasma, urine, and feces [23]. Because circulating parasitic DNA is distributed systemically, it can be detected in multiple specimen types and may reduce the need for repeated stool or urine sampling in low-prevalence settings.

#### 2.9.1. Sample Preparation for DNA Extraction

Stool samples were centrifuged before DNA extraction to enhance detection accuracy. Centrifugation pelleted parasitic cells and cellular debris, concentrating parasitic DNA while removing soluble PCR inhibitors and other contaminants that could interfere with downstream amplification, yielding cleaner samples for molecular testing. Stool samples (pea-sized portions) were suspended in 10 mL of phosphate-buffered saline (PBS, pH 7.4) to disintegrate the stool matrix and release parasitic cells. The suspension was vortexed, centrifuged at 5000 rpm for 10 min, and divided into aliquots for storage at −20 °C until further analysis. This protocol ensured the preservation of parasitic DNA and minimized sample degradation.

#### 2.9.2. cfDNA Extraction from the Stool Sample

DNA extraction from stool samples was performed using the ZymoBIOMICS DNA Miniprep Kit (Zymo Research, Irvine, CA, USA) following the manufacturer’s instructions. The centrifuged stool samples were thawed in preparation for DNA extraction. The protocol was as follows: The suspension was suspended in 150 microlitres (μL) of DNA elution buffer and then transferred into separate Eppendorf tubes. Next, 200 microlitres of bio-fluid—cell buffer solution (red) and 20 μL of proteinase K enzyme were added to digest the sample. The sample was subjected to vortexing to obtain a thorough mixture and thereafter incubated at 55 °C for 10 min on a heating block. A precisely measured volume of 420 μL of genomic binding buffer was added to the digested sample and properly mixed. Subsequently, the mixture was transferred to a Zymo-Spin IIC-XL column placed in a collection tube and subjected to centrifugation at a speed of 14,000 rpm for 1 min. The columns were transferred to fresh collection tubes. Then, 400 μL of DNA pre-wash buffer was added to the columns. The columns were spun at 14,000 rpm for 1 min, and the contents of the collection tubes were then discarded. Next, 700 μL of g-DNA wash buffer was added and the mixture was centrifuged at 14,000 rpm for 1 min. After that, the collection tube was emptied once again. Afterwards, 200 μL of g-DNA wash buffer was added and centrifuged at 14,000 rpm for 1 min. The collection tube containing the flow-through was then discarded. The columns were transferred to sterile microcentrifuge tubes, and 50 μL of DNA elution buffer was added. The mixture was kept at room temperature for 5 min and then centrifuged at 14,000 rpm for 1 min to elute the DNA. Subsequently, the columns were disposed of and the g-DNA, which had already been extracted in the micro-centrifuge tubes, was stored at −20 °C until further analysis by cPCR [9].

#### 2.9.3. Amplification of *S. mansoni* DNA by cPCR

Given the high sensitivity of molecular methods, cPCR was used as a confirmatory diagnostic; however, due to cost constraints, testing was restricted to POC-CCA-positive samples. Genomic DNA was extracted from stool, and 28 samples from individuals positive for *Schistosoma* antigen by POC-CCA were selected for analysis. This selective verification introduces partial verification (work-up) bias, as POC-CCA-negative samples were not tested, precluding reliable estimation of sensitivity, specificity, predictive values, and agreement metrics.

PCR targeted the mitochondrial cytochrome c oxidase subunit 1 (*cox1*) gene of *S. mansoni*, a locus widely used in diagnostic and phylogenetic assays (typical amplicon ~250–950 bp, based on the primer design) [24,25]. The species-specific primers, adapted from Sady et al. (2015) [26] and detailed in Table 1, were designed to amplify highly repetitive genomic regions to enhance detection of low-concentration cell-free DNA. Primers were sourced from Inqaba Biotech (Johannesburg, South Africa) based on prior demonstrated efficacy.

cPCR was performed in a 20 μL reaction containing 10 μL OneTaq 2× Master Mix, 1 μL gDNA (10–30 ng/μL), 1 μL each of forward and reverse primers (10 μM), and 7 μL nuclease-free water (Inqaba Biotech, Johannesburg, South Africa). Thermal cycling comprised initial denaturation at 94 °C for 5 min; 35 cycles of 94 °C for 30 s, 50 °C for 30 s, and 68 °C for 1 min; followed by a final extension at 68 °C for 10 min and held at 4 °C. Reactions were run on a Bio-Rad T100 Thermal Cycler.

#### 2.9.4. Agarose Gel Electrophoresis

cPCR amplicons were verified on a 1% agarose gel prepared in 1× TBE containing ethidium bromide. A 10 μL aliquot of PCR product mixed with 2 μL DNA loading dye was loaded alongside a 10 kb DNA ladder and a negative control. Electrophoresis was performed at 120 V for 25 min, and bands were visualized under UV transillumination. The protocol was adapted from [26].

### 2.10. Data Analysis

Data was entered into a Microsoft Excel spreadsheet, processed to eliminate errors, and then transferred to R software (Version 4.31) for analysis. Sex, age, study site, and grade of participants were reported as frequencies and percentages. The POC-CCA test data was recorded in an Excel spreadsheet, with quantitative intensities coded as follows: negative, trace, 1+ and 2+ transformed into numeric values of 0, 1, 2 and 3, respectively. Results from agarose gel electrophoresis were recorded by the researcher and verified by a team of trained molecular biologists.

### 2.11. Ethics

Ethical approval was obtained from the Research Ethics Committee: Human (REC-H) at Nelson Mandela University (Ref: H23-HEA-ENV-001; approval date: 12 April 2023) before data collection. The Eastern Cape Department of Education and school principals also granted permission. The study adhered to the Belmont Report’s principles of respect for persons, beneficence, and justice. Written informed consent was obtained from parents or guardians, with verbal and written assent from participants aged 5–14 years. Participation was voluntary, with the right to withdraw at any time. Non-invasive procedures minimized risks, and findings aimed to inform schistosomiasis control efforts. Equitable participant selection ensured fairness, and data confidentiality was maintained.

## 3. Results

### 3.1. Sociodemographic Characteristics of the Study Population

A total of 759 participants (57.7% male; 42.3% female) were enrolled. Most participants (61.5%) were in grades 4–7, and 38.5% were in grades 0–3. The mean age was 11.0 ± 1.5 years (range: 5–14). Participation was highest in KwaNobuhle (57.8%), followed by Motherwell (20.0%), Despatch (7.9%), and Ibhayi and Kariega (7.1% each) (Table 2).

The prevalence of *S. mansoni* in this study was evaluated using three diagnostic methods: the conventional Kato–Katz technique, the POC-CCA test, and molecular diagnostics via cPCR. Each method yielded varying levels of sensitivity and diagnostic accuracy, particularly in the low-prevalence settings under investigation.

### 3.2. Kato–Katz Method

The Kato–Katz technique detected no *S. mansoni* eggs among the 759 participants, highlighting its limited sensitivity in low-endemic settings where infection intensities fall below microscopy detection thresholds.

### 3.3. POC-CCA Test

In contrast, the POC-CCA test, which detects *S. mansoni* antigens in urine, identified 28 positive cases out of 759 participants, corresponding to a prevalence rate of 3.7% (95% CI: 2.35–5.03%). Among these positive results, the antigen levels were categorized as follows: twenty-three participants (82.1%) exhibited trace levels, three (10.7%) were classified as low intensity (1+), and two (7.1%) demonstrated medium intensity (2+). These findings highlight the POC-CCA test’s superior sensitivity in detecting low-intensity infections, particularly in comparison to the Kato–Katz method. The predominance of trace-level results in the POC-CCA test data suggests a higher sensitivity for detecting subclinical or low-intensity infections. However, the reliability of these trace results warranted further investigation, which was performed using cPCR [12].

### 3.4. Validation of POC-CCA Test Findings Using cPCR for S. mansoni Detection

To further validate the POC-CCA test findings, cPCR was performed on a subset of 28 POC-CCA-positive samples, targeting the *S. mansoni* cox-1 mitochondrial gene. This method detected *S. mansoni* DNA in nine samples, yielding a positivity rate of 32.1% (95% CI: 14.8–49.4%). The relatively wide confidence interval reflects the small sample size used for cPCR analysis. The cPCR findings not only confirmed the presence of *S. mansoni* DNA, but also highlighted the capacity of molecular diagnostics to identify infections that may be missed by both microscopy and antigen detection methods. The use of degenerate primers (ShmF and ShmR), ensured the specificity of the assay. Amplified DNA fragments were approximately 0.766 Kb.

### 3.5. Summary of Diagnostic Methods for S. mansoni Detection

A comparison of the three diagnostic methods used to detect *S. mansoni* infection among school-going children in NMB is presented in Table 3. The Kato–Katz method identified no cases of *S. mansoni*. The POC-CCA test detected antigens in 3.7% (28/751) of participants. Among the 28 POC-CCA-positive samples that underwent cPCR confirmation, PCR positivity varied by antigen intensity: of the 23 trace-positive samples, 4 (17.4%) were PCR-confirmed; of the 3 samples with 1+ intensity, 2 (66.7%) were PCR-confirmed; and both samples with 2+ intensity (100%) were PCR-confirmed. The overall confirmation rate by cPCR among POC-CCA-positive samples was 32.1% (9/28). Trace results accounted for 82.6% (23/28) of all POC-CCA-positive cases.

## 4. Discussion

This study aimed to determine the prevalence of *S. mansoni* among school-going children in Nelson Mandela Bay, a vulnerable population at higher risk of infection due to frequent exposure to contaminated water sources [3]. By employing a range of diagnostic techniques, this study sought to address the limitations of conventional tools, particularly in low-endemic settings, to improve detection and provide more accurate prevalence estimates [3].

In the present study, no cases of *S. mansoni* were detected using the Kato–Katz method. This finding supports the well-documented challenges of diagnosing low-intensity infections in low-prevalence settings using traditional microscopy [27,28,29,30]. While Kato–Katz is known for its specificity, its sensitivity is limited by small sample volumes and the day-to-day variability in egg excretion, which is particularly problematic in cross-sectional studies like this one, where samples are collected at a single time point [27]. However, the use of additional diagnostic techniques helped to confirm and strengthen the overall accuracy of our findings [29].

This reported prevalence of *S. mansoni* by Kato–Katz in NMB was considerably lower than rates reported in other Sub-Saharan African countries, such as Madagascar, Guinea, and Tanzania, where prevalences range from 9.5% to 13.2% [31]. This finding aligns with the generally lower prevalence reported in South Africa and may be attributable to local environmental changes, such as altered rainfall patterns that have reduced the availability of transmission sites within the study area [4,16]. A previous study in KwaZulu-Natal among preschool-aged children reported a prevalence of 0.9%, further supporting the pattern of low endemicity in the region [3].

The point-of-care circulating cathodic antigen (POC-CCA) test demonstrated higher sensitivity than the Kato–Katz method, with a detected prevalence of 3.7%. This is consistent with evidence that POC-CCA identifies higher prevalence in low-endemic settings than microscopy [32,33]. However, its specificity remains a limitation. Cross-reactivity with other helminths (e.g., hookworm, *Ascaris lumbricoides*), antigenic similarity to host Lewis-X structures, and host factors such as diabetes and haematuria may contribute to false-positive results, particularly for trace readings [34,35,36,37,38]. Specificity as low as 62% has been reported in low-endemic settings [11] and performance may be further influenced by sample preservation, which is relevant in field-based studies. Evidence on the influence of hookworm infection remains inconclusive [35,39].

Despite these limitations, POC-CCA has operational advantages, including improved compliance, low cost, and rapid antigen clearance following treatment, supporting its use in surveillance [27,33]. However, its utility for quantifying infection intensity is limited, as the semi-quantitative scale (trace, 1+, 2+) has not been validated against worm burden or egg output in low-transmission settings. In low-endemic contexts such as NMB, positive or trace POC-CCA results should therefore be interpreted cautiously, as they may indicate possible *S. mansoni* presence, but do not confirm active infection without molecular confirmation.

To address the specificity concerns of the POC-CCA test, this study employed circulating cfDNA detection via cPCR as a confirmatory method on all POC-CCA-positive samples. DNA-based assays are recognized for their reliability in low-endemicity settings. Of the 28 POC-CCA-positive samples analyzed, 9 (32.1%) were confirmed positive for *S. mansoni* DNA by cPCR. This finding aligns with prior investigations, where PCR has consistently identified a higher proportion of infections than Kato–Katz [40]. The lower rate of confirmation by PCR relative to POC-CCA suggests that a portion of the POC-CCA-positive results may represent false positives, potentially due to the cross-reactivity factors discussed above. While cPCR is highly sensitive, its application in resource-limited field conditions is constrained by the cost and infrastructure requirements for thermocyclers, reinforcing the utility of the POC-CCA test as a practical alternative for surveillance [41]. The cPCR results also revealed a higher infection rate among males, a pattern attributed to increased water contact through activities such as swimming and fishing, which is consistent with studies from Nigeria and Senegal [42,43].

The key finding is evidence of ongoing *S. mansoni* transmission in NMB despite zero detection by microscopy. The Kato–Katz method indicated zero prevalence, while POC-CCA suggested 3.7% with uncertain specificity. cPCR confirmed 32.1% (9/28) of POC-CCA-positive samples, providing the first molecular evidence of transmission. However, most positives were trace (82.1%), with a high non-confirmation rate (67.9% overall; 82.6% of trace), consistent with known limitations of POC-CCA in low-prevalence settings (e.g., cross-reactivity). We therefore recommend interpreting trace results as indeterminate pending PCR confirmation and using a two-step approach: POC-CCA screening followed by PCR confirmation to balance sensitivity and specificity.

The recent schistosomiasis outbreak in Tzaneen (2025), which affected over 150 learners, highlights the dynamic and fluctuating nature of transmission, emphasizing the need for strengthened surveillance systems and responsive public health capacity to detect and manage such events [44]. This event emphasizes the urgent need to integrate advanced diagnostic approaches, such as POC-CCA testing and cPCR, into routine surveillance frameworks to improve sensitivity, enable early detection of low-intensity infections, and support timely, evidence-based interventions. It also calls for comprehensive, government-led surveillance programmes coupled with coordinated mass drug administration campaigns and strengthened public health responses to effectively address fluctuating transmission dynamics and advance schistosomiasis control efforts in South Africa.

A key limitation of this study is its cross-sectional design, which precludes assessment of temporal variation in egg excretion, and consequently reduces the sensitivity of single-sample diagnostics. This is particularly relevant for the Kato–Katz thick smear technique, which examines only ~41.7 mg of stool per slide; its small sample volume and non-concentrating nature substantially limit detection, especially in low-intensity infections where egg output is intermittent and sparse. In contrast, concentration methods such as formalin–ethyl acetate sedimentation or decantation permit analysis of larger stool volumes and would likely improve detection rates. However, Kato–Katz remains the standard method in national surveillance programmes in South Africa, and this study aimed to evaluate its performance within that context. Future studies should incorporate concentration techniques to better approximate true prevalence in low-intensity settings. Additionally, the absence of a true positive reference standard constrains robust assessment of diagnostic accuracy and comparative test performance. In the absence of any Kato–Katz-positive cases, it was not possible to directly evaluate the sensitivity of the POC-CCA assay against microscopy-confirmed infection. As noted by Ochodo et al. (2015) [32], while the POC-CCA assay demonstrates high sensitivity in moderate-to-high endemicity settings, its specificity can be variable in low-prevalence areas where trace results are common. The finding that only 32% of POC-CCA-positive samples were confirmed by PCR suggests that specificity may be limited in this context, whereas sensitivity could not be reliably estimated. Future studies should incorporate a panel of confirmed positive cases to better characterize both the sensitivity and specificity of antigen-based tests in low-endemic settings. Additionally, while a comprehensive analysis of co-infections (e.g., *S. haematobium*, soil-transmitted helminths) is beyond the scope of this study, we acknowledge their potential to contribute to cross-reactivity in the POC-CCA assay, as previously reported [34,37]. This will be an important focus of future work.

Future studies should consider molecular screening of all urine and stool samples, rather than only antigen-positive subsets, to provide more accurate estimates of *S. mansoni* prevalence in low-endemic settings. While resource constraints present a challenge, advances in cost-effective molecular techniques and pooled screening approaches may make comprehensive molecular surveillance more feasible in future control programmes. To improve future research and public health interventions, incorporating multiple diagnostic methods, including combining traditional techniques (e.g., Kato–Katz) with advanced approaches (e.g., PCR, POC-CCA), is crucial for obtaining accurate prevalence estimates in low-transmission environments. Routine screening, community education, and enhanced access to molecular diagnostic tools are essential for reducing infection rates and enabling prompt interventions. Finally, a comprehensive understanding of the local snail intermediate hosts remains critical for addressing the ecological dimensions of schistosomiasis transmission.

## 5. Conclusions

In conclusion, the findings of this study highlight the limitations of the Kato–Katz technique, particularly in low-transmission settings, and emphasize the importance of using multiple diagnostic methods to obtain accurate prevalence estimates. The POC-CCA test demonstrated superior sensitivity compared to the Kato–Katz technique, and cPCR provided further confirmation of infections. Although the POC-CCA test is not without limitations in specificity, its utility in detecting low-intensity infections and its cost-effectiveness make it a valuable tool in schistosomiasis control efforts, particularly in resource-limited settings. Combining these methods allows for a more comprehensive understanding of schistosomiasis prevalence and transmission dynamics in low-endemic areas. It must be emphasized, however, that in low-prevalence settings, POC-CCA can only suggest that *S. mansoni* infection may possibly still be present, and its use for quantitative assessment requires further evaluation before it can be applied with confidence in control programmes.

## Figures and Tables

**Table 1:** Genes amplified by cPCR and their respective primer sequences.

Sequence	Primer Name	Primer Sequence	Reference
*cox1* gene	ShbmF SmR	5′-TTTTTTGGTCATCCTGAGGTGTAT-3′F 5′-TGCAGATAAAGCCACCCCTGTG-3′R	[26]

**Table 2:** Demographic characteristics of participants.

Demographic Characteristic	Total Sample Size (*n* = 759)	%
Gender		
Male	438	57.7
Female	321	42.3
Grade		
0–3	292	38.5
4–7	467	61.5
Age (years)		
5–8	76	10
9–14	683	90
Town		
KwaNobuhle	439	57.8
Ibhayi	54	7.1
Kariega	54	7.1
Motherwell	152	20.0
Despatch	60	7.9

Assessment of *S. mansoni* prevalence using Kato–Katz technique, POC-CCA test, and cPCR techniques.

**Table 3:** Diagnostic test results for *S. mansoni* detection among school-going children, comparing stool microscopy (Kato–Katz), antigen detection (POC-CCA test), and molecular diagnostics (cPCR) in a low-prevalence setting.

Test Method	Number of Participants Tested	Positive Cases (n)	Prevalence (%)
Kato–Katz (stool microscopy)	759	0	0
POC-CCA test (antigen detection)	759	28	3.7
cPCR (cox-1 gene)	28 (CCA-positive samples)	9	32.1 (of CCA-positive samples)

## Data Availability

Data is unavailable due to privacy and/or ethical restrictions.

## Abbreviations

The following abbreviations are used in this manuscript:

Diagnostic Tests and Techniques	
cfDNA	cell-free DNA
cPCR	conventional polymerase chain reaction
DNA	Deoxyribonucleic acid
gDNA	genomic DNA
PCR	Polymerase chain reaction
POC-CCA TEST	Point-of-care circulating cathodic antigen (also referred to as POC-CC assay)
PPV	Positive predictive value
Biological and Medical Terms	
CCA	Circulating Cathodic Antigen
cox1	cytochrome c oxidase subunit 1 (a gene)
EPG	Eggs per gram
NPV	Negative predictive value
Organizations and Institutions	
ECDOE	Eastern Cape Department of Education
NMB	Nelson Mandela Bay
REC-H	Research Ethics Committee: Human
Units of Measurement	
bp	base pair(s)
g	gram(me)
Kb	kilobase
km^2^	square kilometres
M	Marker (as in DNA ladder lane)
mg	milligram
minute(s)	minute(s)
ml	millilitre(s)
mm	millimetre(s)
ng/μL	nanograms per microliter
rpm	revolutions per minute
V	volts
μL	microlitre(s)
μM	micromolar
°C	degrees Celsius
Statistical Terms	
CI	Confidence interval
κ	Cohen’s Kappa (kappa statistic)
n	sample size/subset size
Other	
ID	Identifier/identification
PBS	Phosphate-buffered saline
QGIS	Quantum Geographic Information System (software)
Ref	Reference number
TBE	Tris Boric Ethylene Diamine Tetra-acetic Acid (buffer)
UV	Ultraviolet
